# Implementation of neck/shoulder exercises for pain relief among industrial workers: A randomized controlled trial

**DOI:** 10.1186/1471-2474-12-205

**Published:** 2011-09-21

**Authors:** Mette K Zebis, Lars L Andersen, Mogens T Pedersen, Peter Mortensen, Christoffer H Andersen, Mette M Pedersen, Marianne Boysen, Kirsten K Roessler, Harald Hannerz, Ole S Mortensen, Gisela Sjøgaard

**Affiliations:** 1 Institute of Sports Science and Clinical Biomechanics, University of Southern Denmark, 5320 Odense M, Denmark; 2 National Research Centre for the Working Environment, Lersø Parkallè 105, 2100 Copenhagen Ø, Denmark; 3 Department of Exercise and Sport Sciences, Faculty of Science, University of Copenhagen, 2200 Copenhagen N. Denmark; 4 Department of Occupational Health, Bispebjerg University Hospital, 2400 Copenhagen NV, Denmark

## Abstract

**Background:**

Although leisure-time physical activity is important for health, adherence to regular exercise is challenging for many adults. The workplace may provide an optimal setting to reach a large proportion of the adult population needing regular physical exercise. This study evaluates the effect of implementing strength training at the workplace on non-specific neck and shoulder pain among industrial workers.

**Methods:**

Cluster-randomized controlled trial involving 537 adults from occupations with high prevalence of neck and shoulder pain (industrial production units). Participants were randomized to 20 weeks of high-intensity strength training for the neck and shoulders three times a week (n = 282) or a control group receiving advice to stay physically active (n = 255). The strength training program followed principles of progressive overload and periodization. The primary outcome was changes in self-reported neck and shoulder pain intensity (scale 0-9).

**Results:**

85% of the participants followed the strength training program on a weekly basis. In the training group compared with the control group, neck pain intensity decreased significantly (-0.6, 95% CI -1.0 to -0.1) and shoulder pain intensity tended to decrease (-0.2, 95% CI -0.5 to 0.1, P = 0.07). For pain-cases at baseline (pain intensity > = 3) the odds ratio - in the training group compared with the control group - for being a non-case at follow-up (pain intensity < 3) was 2.0 (95% CI 1.0 to 4.2) for the neck and 3.9 (95% CI 1.7 to 9.4) for the shoulders.

**Conclusion:**

High-intensity strength training relying on principles of progressive overload can be successfully implemented at industrial workplaces, and results in significant reductions of neck and shoulder pain.

**Trial registration:**

NCT01071980.

## Background

Musculoskeletal disorders comprise a major burden on individuals and public health systems in North America and Europe [1]. Neck and shoulder pains are among the most frequent health complaints among adults [2,3]. Physical workplace factors such as repetitive work tasks, static contractions, and tiring postures are related to neck and shoulder pain [4].

Studies have evaluated different types of physical exercise for treating neck and shoulder pain [5-8]. While moderate to strong evidence for the effectiveness of strength training for relieving neck pain among office workers exists [9-11], evidence lacks among other occupational groups. Laboratory technicians - commonly exposed to high levels of strain in the neck and shoulders due to prolonged static loadings - show high prevalence of neck and shoulder pain [12,13]. Based on previous research among office workers, investigating the effect of strength training on neck and shoulder pain among laboratory technicians is therefore relevant.

A British health survey reported that among the general population only 37% of men and 24% of women fulfilled public recommendations of physical activity [14]. Thus, regular physical exercise is challenging for many people. In consequence, low adherence to exercise programs can negatively affect the outcome of randomized controlled trials, even in high quality studies [7]. The major reason for not adhering to physical exercise is ''lack of time'' [15]. Thus, workplace interventions with physical exercise during work hours and together with colleagues may reach people with low motivation for leisure physical exercise.

The present study has two major aims: Firstly, to evaluate the effect of strength training intervention at the workplace on non-specific neck and shoulder pain among industrial workers. Secondly, to describe the implementation process and adherence to the program.

## Methods

### Study design

**Figure 1:**
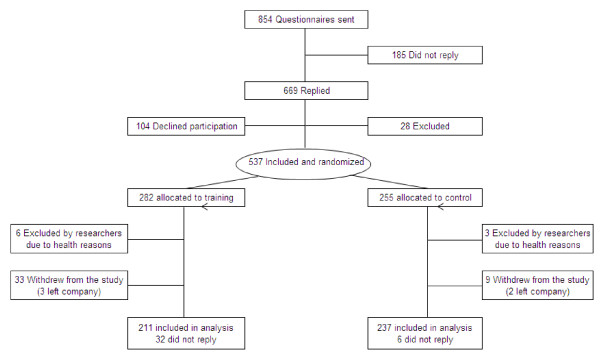
**Flow of participants throughout the intervention**.

A cluster randomized controlled trial was performed in Copenhagen, Denmark. We recruited employees from two large industrial production units - a private sector company specialized in creating bio-industrial products by using enzymes (A) and a public sector company specialized in production of vaccines and control of infectious diseases (B) - in February 2009. At both companies, the daily work of laboratory technicians consisted of repetitive tasks, such as pipetting work, preparing vial samples for analysis, and data processing on a computer including mouse work - all tasks that require precision in work and may result in extended periods of time spent in static working postures [12,13]. The procedure of recruitment is outlined in Figure 1.

Altogether 854 employees were invited to participate in the study. We e-mailed the prospective participants a short introduction and invitation text, together with a link to an internet-based questionnaire. Exclusion criteria were pregnancy and serious health conditions such as previous trauma or injuries, life-threatening diseases and cardiovascular diseases.

In total, 669 replied to the questionnaire, out of which 73 declined to participate, 31 did not answer to the question concerning participation and 28 were excluded due to the above exclusion criteria. Thereby 537 participants were included in the study, and randomly assigned to either a training or control group.

A priori power analysis showed that a sample size of 120 participants in each group would provide a power of 80% to detect a 15% change in pain. At an estimated dropout or loss to follow-up of 20%, the minimally required number of participants in each group should be 150.

We informed all participants about the purpose and content of the project and they gave written consent to participate in the study, which conformed to the Declaration of Helsinki and was approved by the local ethical committee (HC2008103).

### Randomization

The employees who agreed to volunteer for the study were randomized at the cluster-level [16] into either a training group or a control group. To help ensure comparability of the training and control groups, we stratified departments into 14 strata according to the following nested criteria: company, type of work task, size of department. Strata were formed to achieve the above mentioned balances (see Table 1).

**Table 1:** Number of participants within the 14 different strata's divided into the two groups.

	TRAINING GROUP			CONTROL GROUP		
**Stratum**	**Participants (N)**	**Clusters (N)**	**Cluster size (range)**	**Participants (N)**	**Clusters (N)**	**Cluster size (range)**

A	44	7	3-12	58	8	1-15
B	24	3	1-12	27	3	8-11
C	12	3	1-8	10	1	10
D	5	1	5	7	1	7
E	6	1	6	4	1	4
F	9	2	3-6	10	2	3-7
G	36	3	1-27	28	3	5-12
H	.	.	.	4	1	4
I	60	3	2-37	19	2	9-10
J	.	.	.	33	1	33
K	23	1	23	.	.	.
L	24	1	24	40	1	40
M	34	4	4-11	13	2	3-10
N	5	1	5	2	1	2

Total	282	30		255	27	

The number of clusters and the range of the cluster size are specified for each stratum.

Strata were labeled alphabetically and clusters were numbered consecutively within strata. In total, 57 clusters were defined. A statistician - who was blinded to the identity of the strata and clusters - assigned the clusters within each stratum by simple random allocation to either the training or the control group. The consecutive numbers of the clusters within each stratum were written on pieces of paper and drawn from an opaque, tossed plastic bag. To minimize imbalance over several strata with odd numbers of clusters, these strata were paired, and clusters were alternately allocated to either training or control, the first cluster being allocated to either training or control depending on the flip of a coin. Thus all clusters had the same chance of being allocated to the training group while minimizing any biases.

As the clusters inherently contain different number of individuals, a cluster randomization will most of the time result in unequal group sizes. In consequence, 282 employees were allocated to the training group and 255 employees to the control group. Out of 282 employees in the training group, 211 (75%) replied to the follow-up questionnaire. In the control group, 93% replied to the follow-up questionnaire (237 out of 255 employees) (Figure 1).

### Description of Intervention

A high priority was to implement the training program in a way that would ensure high adherence and enable the workplaces to carry on training after the cessation of the study.

**Figure 2:**
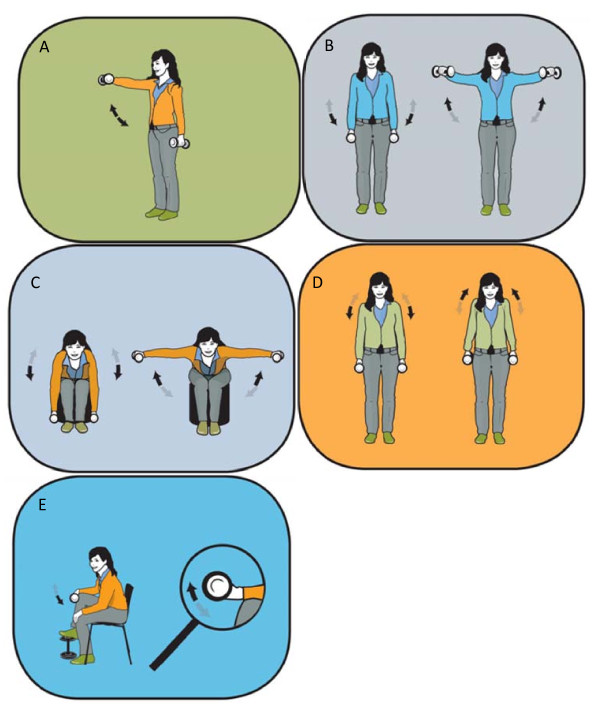
**The five training exercises used in the present study**. A) Front raise, B) lateral raise, C) reverse flies, D) shrugs, and E) wrist extension.

**Figure 3:**
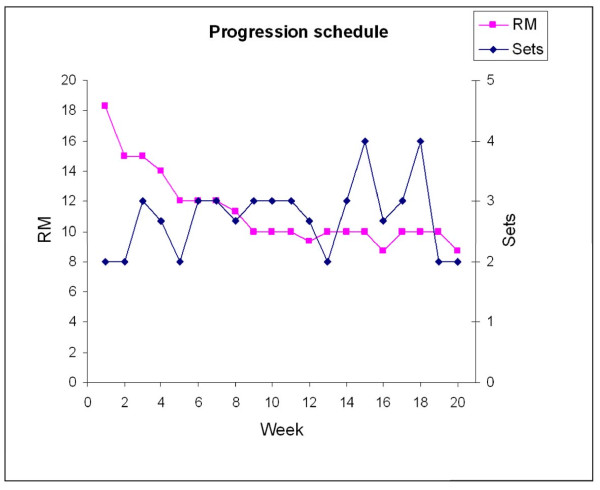
**Progression of relative intensity, i.e. repetition maximum (RM), and number of sets per exercise session throughout the 20 week training period**. Notice that RM was decreased, i.e. the relative intensity increased, while the number of sets was slightly increased, but in an undulating fashion, during the training period. Data points represent weekly mean values of all sets and exercises from the training diaries of the training group (n = 282).

The intervention took place over a 20-week period with questionnaires sent out in January 2009 and June 2009. The participants in the training group were allowed to use a total of one hour a week during work hours for the specific training program. Experienced instructors introduced the program in small groups of approximately 5-15 colleagues. After the introduction the subjects were allowed to train on individual basis or in self organized groups. The training group performed high-intensity specific strength training locally for the neck and shoulder muscles with 4 different dumbbell exercises (A-D, Figure 2) and 1 exercise for the wrist extensor muscles (E, Figure 2). Andersen and coworkers have previously shown a high muscle activity and specificity of the neck/shoulder muscles during similar exercises in both patients with chronic neck pain [17] and healthy individuals [18]. The training regime consisted of three sessions per week, each lasting 20 minutes. During the intervention period the training load was progressively increased according to the principle of progressive overload [19] and both linear (week 1-12) and undulating periodization (week 13-20) strategies were used throughout the training program [20,21] (Figure 3). Periodization is the process of varying a training program at regular time intervals to bring about optimal gains in physical performance. In "linear periodization" training load is gradually increased over time while training volume (total number of repetitions) is decreased. In "undulating periodization" the manipulation of training volume and training load is on a weekly basis; light and heavy weekly training days. After two introductory training sessions - where the participants learned to perform the exercises with correct technique and appropriate load - relative loadings were progressively increased from 15 repetitions maximum (RM; ~70% of maximal intensity) at the beginning of the training period to 8-12 RM (~75-85% of maximal intensity) during the later phase (Figure 3).

The strengthening exercises were performed using consecutive concentric and eccentric muscle contractions with slow to moderate lifting velocity previously shown to reduce neck and shoulder pain in office workers with trapezius myalgia [22].

Experienced exercise instructors were present every other training session. The sessions with supervision were scheduled with assistance from the respective departments to fit into daily working routines. The remaining training sessions were openly planned, meaning that the participants were able to train whenever it matched their daily work activities. Prior to the intervention, we developed a training manual with specific focus on implementation of physical activity at the workplace. All supervisors were educated in accordance to this manual to ensure a standardized supervision with focus on *lifting technique, loading adjustments, exercise/training modifications due to possible training induced soreness/pain, and motivation/barriers towards training*.

We established a hotline with a physiotherapist for participants to consult in case of adverse events - e.g. unexpected joint pain or neck strain - due to training.

The training locations were placed as close as possible to the actual work station for the respective departments/clusters. In practice, we established training locations in store rooms, broad corridors, and conference rooms (11 locations in total). All locations were equipped with a training poster, a clock, chairs, 2 pairs of lifting straps and dumbbells (pairs of 1-25 kg).

The participants in the training group were taught to register the training load for each training session in a personal logbook during the 20 week intervention period. Participants in the control group received advice to stay physically active and were consulted once a week by a supervisor during the 20-week period. After the 20 week intervention period, the control group was offered an equivalent 20 week training period - i.e. 1 hour a week during work hours.

#### Adherence

We defined adherence based on follow-up questionnaire replies on training frequency. The reply options given in the questionnaire were: "2-3 times per week", "1-2 times per week", "1 time per week", "2-3 times per month", "never" and "withdrew from intervention". Regular adherence was defined as participating at least once a week during the 20 week intervention [9].

### Primary outcome measure

#### Neck and shoulder pain intensity

Musculoskeletal symptoms were reported according to a modified version of the Nordic questionnaire on trouble (ache, pain, or discomfort) in the neck and shoulders during the last 7 days [23]. The intensity of pain in the neck and shoulder were rated subjectively on a scale ranging from 0-9 in the questionnaire, where 0 indicated "no pain at all" and 9 indicated "worst possible pain". The following questions were asked: "What degree of pain or discomfort have you experienced in [body part] during the last seven days?" with [body part] replaced first by neck, then by the left shoulder, and then by the right shoulder.

Subsequently, cases were defined as those who scored 3 or more on the 0-9 scale [24]. Non-cases were defined as those who scored from 0 to 2 on the 0-9 scale [24].

### Statistical analyses

All data were analyzed in accordance with the intention to treat principle. We used the GLIMMIX procedure of SAS version 9.2. We performed analysis of variance to model change in pain during the last seven days first in the neck and then in the shoulders. To investigate the effect of the intervention on rehabilitation, first with regard to neck pain and then with regard to shoulder pain, we estimated the odds ratio, with a 95% confidence interval, (training versus control group) of being free of pain (pain intensity during the last week < 3) at follow-up, among cases (pain intensity > = 3) at baseline. To investigate the effect on prevention of pain development, we estimated the odds ratio (training versus control group) of being in pain at follow-up, among those who were non-cases at baseline. Intra-cluster correlations were handled by the inclusion of a random cluster effect. In the shoulder pain analysis, each person contributed with two observations (one for each shoulder). A random person effect was included to deal with intra-person correlations. Variance components correlation structures were assumed. All analyses were controlled for gender.

The level of significance was set to p < 0.05. Baseline results are presented as mean (SD) and changes from baseline to follow-up as means (95% confidence intervals) unless otherwise stated.

## Results

The participants of the training and control groups matched at baseline for demographics, musculoskeletal pain symptoms, and work-related characteristics (Table 2). However, the control group had a higher proportion of men than the training group. We controlled for this difference in the analysis below. Among those who declined to participate at baseline, the proportion of men was significantly higher than among those who agreed to participate (p < 0.001). Further, pain intensity was lower in the neck (p < 0.05) and right shoulder (p < 0.001) among decliners compared with those who participated in the intervention. No differences were observed in work exposure between those who declined and those who agreed to participate at baseline (Table 2).

**Table 2:** Baseline characteristics of the participants in the control group (n = 255), training group (n = 282) and among decliners (n = 73).

	Agreed to participate		Declined to participate	Agreers vs. decliners
	**Control**	**Training**		**P-value**
Demographics:				
Age, year	42 (10)	42 (11)	42 (12)	0.67
Height, cm	170 (8)	168 (8)	173 (9)	0.001
Weight, kg	73 (14)	70 (14)	72 (13)	0.83
Body Mass Index, kg^.^m^-2^	25 (5)	25 (4)	24 (4)	0.04
Women (%)	80%	89%	67%	< 0.001
More than 30 days with pain previous year (% of participants):				
Neck	31%	34%	17%	< 0.01
Right shoulder	20%	27%	6%	< 0.001
Left shoulder	13%	17%	11%	0.39
Pain intensity of 3 or more during previous week (% of participants):				
Neck	31%	34%	20%	< 0.05
Right shoulder	27%	28%	8%	< 0.001
Left shoulder	17%	17%	14%	0.56
Percentage of participants spending more than half of total work time:				
Sitting	87%	83%	92%	0.11
Standing	37%	41%	42%	0.09
Bend forward without arm- or hand- support	9%	11%	14%	0.38
Twisting or bending the back	23%	32%	21%	0.20
Hand at shoulder height or higher	1%	0%	3%	0.05
Performing physical strenous work	10%	14%	10%	0.49
Bent neck	24%	29%	19%	0.17
Hand twisted or flexed	28%	33%	30%	0.94
The same finger movements several times a minute	57%	65%	62%	0.91
The same arm movements several times a minute	34%	38%	33%	0.60
Static work posture	48%	51%	58%	0.19
Kneeling	2%	0%	3%	0.26
Other work-related characteristics:				
Weekly working hours	35 (8)	35 (8)	35 (9)	0.85
Years working in the same type of job	15 (11)	16 (12)	12 (12)	0.07

Baseline characteristics are given as mean (SD).

### Adherence

Adherence to the training program was high with 63% participating 2-3 times per week, 15% participating 1-2 times per week and 7% participating 1 time per week. Thus, regular adherence was achieved by 85% of the participants. There was no difference in training adherence between cases and non-cases.

### Training progression

On average, participants more than doubled their training loads during the 20 week intervention - e.g. from 8 ± 4 kg to 21 ± 7 kg in shrugs. Similar relative improvements were observed in the other exercises. Average weekly progression in sets and RM loadings are given in Figure 3.

### Overall effect of training

On average, the overall intensity of neck pain at baseline was 1.8 ± 2.0 in the training group and 1.8 ± 2.2 in the control group (median values [25th and 75th percentiles] were 1.0 [0.0 and 3.0] and 1.0 [0.0 and 3.0] in the training and control groups, respectively). At follow-up, the overall intensity of neck pain was reduced by 49% in the training group and 17% in the control group.

For the main analysis which included all participants - i.e. both cases and non-cases - analysis of variance controlled for gender showed a significant group by time effect for pain in the neck (p < 0.001) and a tendency for the shoulders (P = 0.07). Compared with the control group, pain intensity in the neck decreased significantly (-0.6, 95% confidence interval -1.0 to -0.1) in the training group, and pain intensity in the shoulder tended to decrease (-0.2, 95% confidence interval -0.5 to 0.1).

### Rehabilitative effect of training

Table 3 shows for cases and non-cases separately, pain intensity in the neck and shoulder at baseline and follow-up. For the participants defined as cases at baseline, the odds ratio - in the training group compared with the control group - for being non-cases at follow-up was 2.0 (95% confidence interval 1.0 to 4.2) for the neck and 3.9 (95% confidence interval 1.7 to 9.4) for the shoulder.

**Table 3:** Pain intensity in the neck and shoulder at baseline and follow-up for cases and non-cases, separately.

			n	Baseline	Followup
Cases	Neck	Control	77	4.6 (1.8)	2.9 (2.3)
		Training	95	4.7 (1.6)	1.8 (1.9)
	R shoulder	Control	69	4.7 (1.8)	2.5 (2.6)
		Training	76	4.8 (1.7)	1.4 (1.7)
	L shoulder	Control	43	5.0 (1.8)	2.2 (2.6)
		Training	46	4.5 (1.5)	0.9 (1.3)
Non-cases	Neck	Control	175	0.5 (0.7)	0.8 (1.5)
		Training	182	0.6 (0.8)	0.5 (1.3)
	R shoulder	Control	183	0.4 (0.7)	0.5 (1.2)
		Training	200	0.6 (0.8)	0.5 (1.2)
	L shoulder	Control	209	0.4 (0.7)	0.5 (1.1)
		Training	231	0.4 (0.7)	0.4 (1.0)

Pain intensity is given as mean (SD).

### Preventative effect of training

For the participants defined as non-cases at baseline, the odds ratio - in the training group compared with the control group - for being cases at follow-up was 0.6 (95% confidence interval 0.2 to 1.5) for the neck and 0.6 (95% confidence interval 0.3 to 1.3) for the shoulder.

On an exploratory basis, we tested the statistical model with workplace (A and B) as a factor. This analysis showed that there was no effect of workplace on the neck and shoulder pain outcome.

## Discussion

Our study showed that specific strength training reduced the overall level of neck pain among industrial workers. Among cases in the training group, the decrease in pain intensity of approximately 3 on a scale of 0-9 was considered clinically important [25,26].

As shown by Table 2, the industrial workers in the present study were highly exposed to known risk factors for development of musculoskeletal pain. Thus, a high percentage of daily activities were performed with static work postures and bent neck (Table 2). In spite of the high physical occupational strain, high intensity strength training was effective in reducing neck pain in this job group.

Our program, which effectively reduced neck and shoulder pain in laboratory technicians, involved dynamic muscle contractions with a high intensity (8-15 RM) and a high volume (6-12 sets per session) performed in a progressive manner with both linear and undulating periodization strategies throughout the training program [20,21] (Figure 3). A variety of strength training protocols for decreasing neck pain have been described in the literature - i.e. low intensity training [7,27], high intensity concentric contractions [28], high intensity isometric contractions [6,8], low total training volume [6], and non-periodized training [6,8,27,28]. The referred studies had in common that the effect of strength training was examined in selected symptomatic groups. In contrast, the finding of the present study is translational to the working population with repetitive work exposure and a high prevalence of neck and shoulder pain symptoms.

Chronic neck pain symptoms are known to display seasonal variation, worsening in the autumn and decreasing in the spring [29]. Thus, a general decrease in neck pain symptoms could be expected as the study ran from January to June. Despite the well-known seasonal variation and thus a decrease of pain in the control group, we found a significantly better rehabilitative effect of strength training than control (OR 2.0).

In contrast to the evidence on neck pain, only few high quality studies on training have been able to provide evidence for the effectiveness on shoulder symptoms [30,31]. Among workers with shoulder pain at baseline, the odds ratio for being a non-case - i.e. having a pain intensity less than 3 at follow-up - were 3.9 in the training group compared with the control group. Thus, the present protocol provides a promising tool for treating pain in the shoulders among industrial workers.

The preventative effect of training on development of pain symptoms - i.e. for non-cases at baseline - was negligible in the present study regardless of body part. A previous study showed that strength training performed for a one-year period had a small but statistically significant preventative effect on development of neck-shoulder symptoms among office workers [30]. Thus, 20-week intervention duration may be insufficient to detect similar preventative effect.

A major strength of the present study is that regular adherence to training was achieved by 85% of the participants. Thus, the high adherence allowed us to investigate the actual effect of the intended intervention. Training facilities located within meters from the work station combined with a training program that could be conducted without changing clothes or subsequently needing a shower, allowed the participants to train whenever it matched into daily work activities. These factors combined with high availability of well educated training instructors may explain the high adherence. Overall, the high adherence shows that the workplace adjusted intervention model used in the present study can be successfully implemented at industrial workplaces.

Adverse events due to overload or incorrect strength training technique were minor and transient. Altogether, fifteen participants consulted our physiotherapist solely due to complaints from previous musculoskeletal injuries. All fifteen participants completed the 20-week training intervention, and showed - based on the baseline and follow-up questionnaire - a reduction in pain symptoms. However, four participants in the training group, who did not consult our physiotherapist, withdrew from the study and gave musculoskeletal pain as their reason. Thus, training may have provoked an adverse effect in the four participants mentioned, corresponding to approximately one percent of the participants of the training group.

We also compared baseline characteristics of those agreeing and declining, respectively, participation in the study. This comparison (Table 2) showed that employees with higher pain levels were more interested in participating than those with lower pain levels. Thus, pain *per se *is not a hindrance for participating in intensive strength training, rather on the contrary.

## Conclusion

In conclusion, high-intensity strength training relying on principles of progressive overload can be implemented at industrial worksites with high adherence, and results in significant and clinically important reductions of neck and shoulder pain.

## Competing interests

The authors declare that they have no competing interests.

## Authors' contributions

MKZ, GS, OSM, LLA and MTP were responsible for the research design. MKZ drafted the paper, and all co-authors made significant contributions to drafting the protocol. All authors have read and approved the final manuscript.

## Pre-publication history

The pre-publication history for this paper can be accessed here:

http://www.biomedcentral.com/1471-2474/12/205/prepub

## References

[B1] Punnett L Wegman DH Work-related musculoskeletal disorders: the epidemiologic evidence and the debate J Electromyogr Kinesiol 2004 12 13 23 10.1016/j.jelekin.2003.09.015 14759746

[B2] Bot SD van der Waal JM Terwee CB van der Windt DA Schellevis FG Bouter LM Dekker J Incidence and prevalence of complaints of the neck and upper extremity in general practice Ann Rheum Dis 2005 12 118 123 10.1136/ard.2003.019349 15608309 PMC1755209

[B3] Larsson B Sogaard K Rosendal L Work related neck-shoulder pain: a review on magnitude, risk factors, biochemical characteristics, clinical picture and preventive interventions Best Pract Res Clin Rheumatol 2007 12 447 463 10.1016/j.berh.2007.02.015 17602993

[B4] National Research Council Institute of medicine Musculoskeletal disorders and the workplase - Low back and upper extremities 2001 Washington D.C.: National Research Council; Institute of medicine ISBN-10: 0-309-07284-0

[B5] Ahlgren C Waling K Kadi F Djupsjobacka M Thornell LE Sundelin G Effects on physical performance and pain from three dynamic training programs for women with work-related trapezius myalgia J Rehabil Med 2001 12 162 169 11506214

[B6] Hagberg M Harms-Ringdahl K Nisell R Hjelm EW Rehabilitation of neck-shoulder pain in women industrial workers: a randomized trial comparing isometric shoulder endurance training with isometric shoulder strength training Arch Phys Med Rehabil 2000 12 1051 1058 10.1053/apmr.2000.7582 10943754

[B7] Viljanen M Malmivaara A Uitti J Rinne M Palmroos P Laippala P Effectiveness of dynamic muscle training, relaxation training, or ordinary activity for chronic neck pain: randomised controlled trial BMJ 2003 12 475 12946968 10.1136/bmj.327.7413.475 PMC188429

[B8] Ylinen J Takala EP Nykanen M Hakkinen A Malkia E Pohjolainen T Karppi SL Kautiainen H Airaksinen O Active neck muscle training in the treatment of chronic neck pain in women: a randomized controlled trial JAMA 2003 12 2509 2516 10.1001/jama.289.19.2509 12759322

[B9] Andersen LL Jorgensen MB Blangsted AK Pedersen MT Hansen EA Sjogaard G A randomized controlled intervention trial to relieve and prevent neck/shoulder pain Med Sci Sports Exerc 2008 12 983 990 10.1249/MSS.0b013e3181676640 18461010

[B10] Groenningsaetter H Hytten K Skauli G Christensen CC Ursin H Improved health and coping by physical exercise or cognitive behavioral stress management training in a work environment Psychology and Health 1992 12 147 163 10.1080/08870449208520016

[B11] Sjogren T Nissinen KJ Jarvenpaa SK Ojanen MT Vanharanta H Malkia EA Effects of a workplace physical exercise intervention on the intensity of headache and neck and shoulder symptoms and upper extremity muscular strength of office workers: a cluster randomized controlled cross-over trial Pain 2005 12 119 128 10.1016/j.pain.2005.03.031 15927388

[B12] Bjorksten MG Almby B Jansson ES Hand and shoulder ailments among laboratory technicians using modern plunger-operated pipettes Appl Ergon 1994 12 88 94 10.1016/0003-6870(94)90069-8 15676954

[B13] David G Buckle P A questionnaire survey of the ergonomic problems associated with pipettes and their usage with specific reference to work-related upper limb disorders Appl Ergon 1997 12 257 262 10.1016/S0003-6870(97)00002-1 9414365

[B14] Allender S Peto V Scarborough P Boxer A Rayner M Physical activity Diet, physical activity and obesity statistics 2006 London: British Heart Foundation 42 66

[B15] Trost SG Owen N Bauman AE Sallis JF Brown W Correlates of adults' participation in physical activity: review and update Med Sci Sports Exerc 2002 12 1996 2001 10.1097/00005768-200212000-00020 12471307

[B16] Campbell MK Elbourne DR Altman DG CONSORT statement: extension to cluster randomised trials BMJ 2004 12 702 708 10.1136/bmj.328.7441.702 15031246 PMC381234

[B17] Andersen LL Kjaer M Andersen CH Hansen PB Zebis MK Hansen K Sjogaard G Muscle activation during selected strength exercises in women with chronic neck muscle pain Phys Ther 2008 12 703 711 10.2522/ptj.20070304 18339796

[B18] Andersen LL Andersen CH Mortensen OS Poulsen OM Bjornlund IB Zebis MK Muscle activation and perceived loading during rehabilitation exercises: comparison of dumbbells and elastic resistance Phys Ther 2010 12 538 549 10.2522/ptj.20090167 20133444

[B19] Kraemer WJ Adams K Cafarelli E Dudley GA Dooly C Feigenbaum MS Fleck SJ Franklin B Fry AC Hoffman JR Newton RU Potteiger J Stone MH Ratamess NA Triplett-McBride T American College of Sports Medicine position stand. Progression models in resistance training for healthy adults Med Sci Sports Exerc 2002 12 364 380 11828249 10.1097/00005768-200202000-00027

[B20] Kraemer WJ Ratamess NA Fundamentals of resistance training: progression and exercise prescription Med Sci Sports Exerc 2004 12 674 688 10.1249/01.MSS.0000121945.36635.61 15064596

[B21] Rhea MR Ball SD Phillips WT Burkett LN A comparison of linear and daily undulating periodized programs with equated volume and intensity for strength J Strength Cond Res 2002 12 250 255 11991778

[B22] Andersen LL Kjaer M Sogaard K Hansen L Kryger AI Sjogaard G Effect of two contrasting types of physical exercise on chronic neck muscle pain Arthritis Rheum 2008 12 84 91 10.1002/art.23256 18163419

[B23] Kuorinka I Jonsson B Kilbom Å Vinterberg H Biering-Sørensen F Andersson G Jørgensen K Standardised Nordic questionnaires for the analysis of musculoskeletal symptoms Appl Ergo 1987 12 233 237 10.1016/0003-6870(87)90010-X 15676628

[B24] Kaergaard A Andersen JH Rasmussen K Mikkelsen S Identification of neck-shoulder disorders in a 1 year follow-up study. Validation Of a questionnaire-based method Pain 2000 12 305 310 10.1016/S0304-3959(00)00261-X 10812260

[B25] Dworkin RH Turk DC McDermott MP Peirce-Sandner S Burke LB Cowan P Farrar JT Hertz S Raja SN Rappaport BA Rauschkolb C Sampaio C Interpreting the clinical importance of group differences in chronic pain clinical trials: IMMPACT recommendations Pain 2009 12 238 244 10.1016/j.pain.2009.08.019 19836888

[B26] Kovacs FM Abraira V Royuela A Corcoll J Alegre L Tomas M Mir MA Cano A Muriel A Zamora J Del Real MT Gestoso M Mufraggi N Minimum detectable and minimal clinically important changes for pain in patients with nonspecific neck pain BMC Musculoskelet Disord 2008 12 43 10.1186/1471-2474-9-43 18402665 PMC2375888

[B27] Randlov A Ostergaard M Manniche C Kryger P Jordan A Heegaard S Holm B Intensive dynamic training for females with chronic neck/shoulder pain. A randomized controlled trial Clin Rehabil 1998 12 200 210 10.1191/026921598666881319 9688035

[B28] Waling K Sundelin G Ahlgren C Jarvholm B Perceived pain before and after three exercise programs--a controlled clinical trial of women with work-related trapezius myalgia Pain 2000 12 201 207 10.1016/S0304-3959(99)00265-1 10692619

[B29] Takala E-P Viikari-Juntura E Moneta GB Saarenmaa K Kaivanto K Seasonal variation in neck and shoulder symptoms Scand J Work Environ Health 1992 12 257 61 1411369 10.5271/sjweh.1580

[B30] Blangsted AK Sogaard K Hansen EA Hannerz H Sjogaard G One-year randomized controlled trial with different physical-activity programs to reduce musculoskeletal symptoms in the neck and shoulders among office workers Scand J Work Environ Health 2008 12 55 65 18427699 10.5271/sjweh.1192

[B31] Ludewig PM Borstad JD Effects of a home exercise programme on shoulder pain and functional status in construction workers Occup Environ Med 2003 12 841 849 10.1136/oem.60.11.841 14573714 PMC1740414

